# In Memoriam: Paolo Cappa

**DOI:** 10.3390/s17112661

**Published:** 2017-11-18

**Authors:** Eduardo Palermo, Stefano Rossi, Fabrizio Patanè, Jeffrey Laut, Maurizio Porfiri

**Affiliations:** 1 Department of Mechanical and Aerospace Engineering, Sapienza University of Rome, 00184 Rome, Italy; eduardo.palermo@uniroma1.it; 2 Department of Economics, Engineering, Society and Business Organization (DEIM), University of Tuscia, 01100 Viterbo, Italy; Stefano.rossi@unitus.it; 3 Niccolò Cusano University, via Don Gnocchi, 00166 Rome, Italy; fabrizio.patane@unicusano.it; 4 Department of Mechanical and Aerospace Engineering, New York University Tandon School of Engineering, Brooklyn, NY 11201, USA; jlaut@nyu.edu

**Keywords:** biomechanics, mechanical measurement, rehabilitation, robotics, thermal measurement

## Abstract

Prof. Paolo Cappa passed away on 26 August 2016, at the age of 59, after a long and courageous fight against cancer. Paolo Cappa was a Professor in Mechanical and Thermal Measurements and Experimental Biomechanics in the Department of Mechanical and Aerospace Engineering of Sapienza University of Rome, where he had also served as the Head of the Department, and a Research Professor in the Department of Mechanical and Aerospace Engineering of New York University Tandon School of Engineering. During his intense, yet short, career, he made several significant scientific contributions within the discipline of Mechanical and Thermal Measurements, pioneering fundamental applications to Biomechanics. He co-founded the Motion Analysis and Robotics Laboratory (MARLab) within the Neurorehabilitation Division of IRCCS Pediatric Hospital “Bambino Gesu”, in Rome, to fuel transitional research from the laboratory to clinical practice. Through collaboration with neurologists and physiatrists at MARLab, Prof. Cappa led the development of a powerful array of novel mechanical solutions to wearable robotics for pediatric patients, addressing dramatic needs for children’s health and contributing to the training of an entire generation of Mechanical Engineering students.

## Prof. Cappa, How We Remember Him: A Leader in Sensors Research in Italy and an Example of a Faculty Member

**Figure 1:**
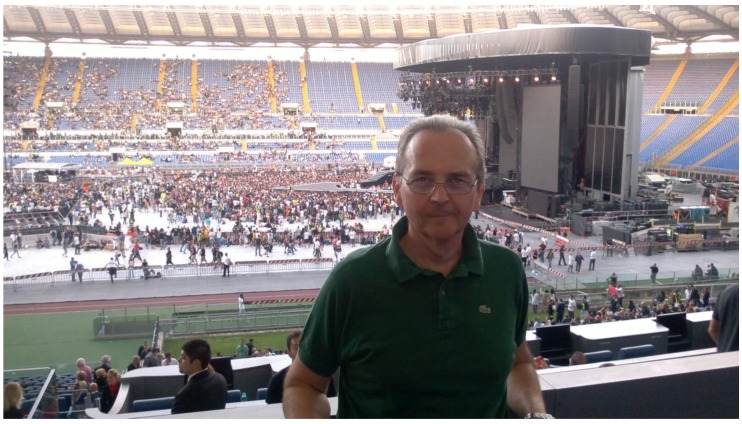
Prof. Paolo Cappa at the Stadio Olimpico of Rome for a concert.

Paolo Cappa (Figure 1) was born in Rome on 13 September 1956. After attending the “Liceo Giulio Cesare”, a renowned high school in Rome, he entered the School of Engineering of Sapienza University in 1975 as a freshman, and graduated *cum laude* in Mechanical Engineering in 1980. Upon graduation, he accepted the offer of a research fellowship from the Department of Mechanical and Aerospace Engineering, and three years later, he was appointed as an Assistant Professor. Through a scholarship awarded by the “Consiglio Nazionale delle Ricerche” as a brilliant young researcher, he started collaborating with Profs. Kenneth MacConnell and Loren Zachary at Iowa State University in 1988.

Within this international collaboration, Prof. Cappa took on a five-month appointment as a visiting scholar in the Department of Aerospace Engineering and Engineering Mechanics of Iowa State University in Ames, IA, in 1989, far from his wife Maria Letizia, married three years earlier, and his two-year-old son Francesco. During this very first period of his career, Prof. Cappa made important contributions to the field of Mechanical and Thermal Measurements, highlighted by his work on the “duplex gage” [1], a sensor for simultaneously measuring strain and temperature, and the evaluation of a methodology to “read” strain gages without a Wheatstone bridge [2].

In 1992, he was promoted to the rank of Associate Professor in the Department of Mechanical and Aerospace Engineering at Sapienza University. He returned to Ames twice more after his promotion: during the summer of 1993, to complete a collaborative effort on multichannel automatic data-acquisition systems for strain gauges [3]; and during the summer of 1996, to finalize a study on the effects of base mounting on force transducers [4]. In 2002, Prof. Cappa was promoted to the rank of (Full) Professor in Mechanical and Thermal Measurement. From 2003 to 2006, he was elected the Head of the Department of Mechanical and Aerospace Engineering at Sapienza University.

In 2007, Prof. Cappa started a new international collaboration with the United States, and this time with a Sapienza alumnus, Prof. Maurizio Porfiri, in the Department of Mechanical and Aerospace Engineering of the Tandon School of Engineering of New York University (NYU). This collaboration contributed to a number of technical and educational achievements and was recognized with the appointment of Prof. Cappa as a Research Professor in the Department of Mechanical and Aerospace Engineering of NYU Tandon.

As a key member of the Italian Steering Committee in Mechanical Engineering, Prof. Cappa led an impressive variety of research projects in Mechanical and Thermal Measurements. His research initiated in what one may call “traditional” Measurement Science, that is, experiments on strain/stress measurement [1,3,4,5,6], but quickly evolved toward the field of Biomechanics, which constitutes the bulk of his career endeavors.

This transition was nurtured by his commitment to children’s health and wellness, through active membership in the clinical engineering service of the “Bambino Gesù” Children’s Hospital in Rome. Among the numerous medical instruments that he was charged with evaluating during his 30-years of experience at the engineering service, particularly remarkable are the characterization of the performance of pulmonary neonatal ventilators and the design of pulmonary simulators [7,8,9,10,11,12], which are critical to ensure the correct level of compliance and breath-triggering threshold for children.

At the beginning of the new millennium, he began a fruitful collaboration with the Pediatric Neuro-Rehabilitation Division of the Children Hospital “Bambino Gesù”. Such a collaboration led to the foundation of the Movement Analysis and Robotics Laboratory (MARLab), where researchers, bioengineers, physiatrists, neurophysiologists, physical therapists, and students have worked together under his caring, visionary, and pragmatic guidance. At MARLab, Prof. Cappa’s research mission was to push the boundaries of Mechanical and Thermal Measurements to contribute to Healthcare. His technical depth and passion for the field enabled Prof. Cappa to contribute several remarkable advancements, in terms of scientific discoveries and enabling engineering technologies that have the potential to transform healthcare.

In the last two decades, his research harmoniously focused on two major subfields: (i) the characterization of existing measurement technologies, along with the design, development, and testing of novel solutions, for Experimental Biomechanics; and (ii) the design, development, and testing of devices for Robot Mediated Therapy (RMT). The technical complexity of these subfields called for a transformative research approach, which Prof. Cappa has put forward to improve efficacy and quality of life of patients, via state-of-the-art technologies and scientifically-principled experiments.

Prof. Cappa was a pioneer in the systematic evaluation of measurement systems in Experimental Biomechanics. Throughout his research, he assessed the metrological characteristics of optoelectronic systems [13,14,15,16], pressure platforms [17], and dynamometers [18], with a particular focus on inertial measurement systems (IMUs), which have constituted one of his primary research topics.

His early studies on IMUs focused on the assessment of angular accelerations and angular velocities of the head, via a helmet instrumented only with linear accelerometers [19,20,21]. The evaluation of head kinematics is a critical aspect for pathologies associated with the vestibular system apparatus. After the addition of gyroscopes to the helmet, he proposed a novel algorithm for estimating head rotations using only accelerometers and gyroscopes, for exploring head compensation mechanisms elicited by body rotations, an important insight within causes of impairment in paretic patients [22].

Shifting the focus to commercial inertial systems, he evaluated the effect of indoor magnetic field disturbances on IMUs used for gait analysis [23]. He also proposed and validated a novel functional calibration procedure to estimate relative rotations between IMUs and body segments where the sensors are placed [24]. These findings paved the way for moving human motion analysis from traditional lab settings to complex unstructured environments, such as those involved in sports. Prof. Cappa was particularly happy to coordinate a study on measuring biomechanical parameters through IMUs during the coaching sessions of elite soccer players of S.S. Lazio [25], the soccer team he supported since he was a child.

Another remarkable contribution by Prof. Cappa to the international research community entailed the estimation of human gait phases using IMUs. Specifically, he proposed a novel distributive algorithm based on Hidden Markov Models capable of detecting gait phases on-the-fly with a minimal computation cost [26,27,28,29].

In the last years of his life, he built on his profound metrological knowledge of IMUs, to establish an empowering array of indices to quantify motor performance in subjects with neurological diseases, such as Multiple Sclerosis [30] and Parkinson’s disease [31,32].

The interest in exploring how robotic technologies could strengthen the medical/rehabilitative field coincided with the foundation of MARLab in 2000. Inspired by the state-of-the-art in rehabilitation tools, such as proprioceptive balance boards or gym equipment modified by therapists, the MARLab started a robotics-centered revolution in instrumentation in Italy, echoing worldwide. Prof. Cappa led the design and evaluation of high-tech devices that would allow reproducible and measurable rehabilitation treatments. Among several important devices, particularly impressive are the robotic solutions for ankle rehabilitation [33,34] and posture treatment [35,36].

The close collaboration with Prof. Allain Berthoz at College de France, led to the realization of RotoBit^3D^ [37,38], an admittance-controlled mechatronic platform designed for the assessment and rehabilitation of subjects with equilibrium impairment. The collaboration with Dr. Hermano Igo Krebs at Massachusetts Institute of Technology resulted in the design of the pediatric version of the ANKLEbot, a wearable robot for ankle rehabilitation [33]. Toward further developing the wearable aspect of robotic rehabilitation solutions, he coordinated an ambitious 3-year project, aimed at designing a modular pediatric knee and ankle exoskeleton, known as “Wake-UP!” [34,39].

Departing from robotics-based physical rehabilitation, in collaboration with Prof. Porfiri at NYU, Prof. Cappa led a new interdisciplinary endeavor on the identification of motivational drivers in rehabilitation and their systematic activation through gaming and citizen science activities. Specifically, the feasibility of enhancing rehabilitation treatment through the inclusion of science learning was demonstrated in [40]. The approach adopted an off-the-shelf haptic device initially intended for gaming, in an effort to increase accessibility to RMT by decreasing cost.

This concept was later solidified in MARLab, where young patients used the low-cost device to simultaneously contribute to a citizen science task and perform rehabilitative exercises [41]. The positive results from these studies, demonstrating the usefulness of science learning and citizen science participation in rehabilitation, fueled more recent efforts in collaboration with NYU in both memory training [42] and physical exercise [43], supported by the National Science Foundation.

Prof. Cappa was profoundly devoted to the international training of Italian students. Thanks to his tireless efforts, hundreds of mechanical engineering students from Sapienza University have had the opportunity to study abroad and gain a mature, global perspective in engineering. As a key member of the Honors Center of Italian Universities (H2CU) in Rome, Prof. Cappa was the architect of a dual degree program with NYU Tandon in Mechanical Engineering and served as its enthusiastic and dedicated coordinator with Prof. Porfiri. This program has contributed to transforming the life of approximately one hundred graduate students, who participated in a unique program, centered around Prof. Cappa’s vision.

Prof. Cappa was recognized for his research at Iowa State University by the Italian National Research Council (1989) and for his remarkable efforts to create international research experiences for Italian students by the Italian Consulate in New York (2013). In 2016, he won the SAPIO award for Research and Innovation in the “Innovation” category with his research on a wearable exoskeleton for children with gait disorders.

Prof. Cappa served as Principal Investigator for numerous research grants. Several of them, supported by the Italian Ministry of Public Education (2006), the Italian Institute of Technology (2009), and the Italian Ministry of Health (2007, 2008, and 2009), were focused on the design and the development of robotic devices for the postural and gait rehabilitation. Two grants funded by the Italian Ministry of Public Education (2012) and the European Community—FP7 (2012) were dedicated to the metrological assessment of optoelectronic systems, electromyography devices, and force transducers that are generally used in clinical routine. With respect to mechanical measurements applied to the industrial field, he served as Principal Investigator of the Sapienza Research Unit for a grant by the Italian Ministry of Education (2004) on the experimental evaluation of the effects induced by vibrations on industrial weighting systems.

Prof. Cappa authored and co-authored over 200-refereed journal and proceeding papers. He wrote three books in Mechanical and Thermal Measurements that have become fundamental references for researchers and students in experimental techniques for industrial and biomedical applications. He mentored more than 100 B.S. and M.S. students and 19 Ph.D. students, many of whom have become researchers and faculty members in Mechanical and Thermal Measurements following his academic example.

Paolo was a caring, humble, and sincere person who was universally respected by his colleagues, students, and friends. A man with a special talent in combining a visionary attitude with a serene personality. We all remember him as a “gentleman”, who best exemplified the profession of a researcher and a faculty member. But also, he was funny, really funny; he had the humor and wit that only very intelligent and gifted people possess, combined with the warm heart and freshness of a teenager starting to explore the world.

After more than one year of his passing away, we still mourn him and miss him very much. He left a large void in the technical community and in the heart of all of us who were very close to him. This void is impossible to fill, but we hope that his exemplary research in healthcare, fatherly dedication to his mentees and students, impeccable demeanor, and visceral humanity will inspire young scholars starting their career in the field of Mechanical and Thermal Measurements.
